# Research progress in imaging detection of brain metastases

**DOI:** 10.3389/fmed.2026.1774662

**Published:** 2026-05-22

**Authors:** Yichen Wang, Chenqi Liang, Wuzhe Liu, Wei Wang, Dongxiang Wang, Daobo Dong, Qingbei Lian

**Affiliations:** 1 School of Clinical and Basic Medicine, Shandong First Medical University, Tai’an, China; 2 School of Radiology, Shandong First Medical University, Tai’an, China; 3 Department of Radiology, Shandong University of Traditional Chinese Medicine Affiliated Hospital, Jinan, China; 4 Department of Neurosurgery, Shandong Second Provincial General Hospital, Jinan, China

**Keywords:** advanced MRI sequence, brain metastases, MRI, multimodal fusion technology, PET

## Abstract

Brain metastasis (BM) is a common and severe complication of advanced cancer, and early, accurate diagnosis is critical to improving patient outcomes. This study comprehensively analyzes the characteristics of various imaging techniques for detecting BM. Conventional magnetic resonance imaging (MRI) remains the primary diagnostic method due to its high soft-tissue resolution, while advanced MRI techniques have improved the detection of small lesions. Computed tomography (CT) is valuable for rapid emergency assessment but is limited by lower soft-tissue resolution and radiation exposure. PET imaging offers unique advantages in functional imaging and treatment response differentiation, though constrained by signal-to-noise ratio limitations. Multimodal fusion techniques (e.g., PET/CT, PET/MRI) integrate anatomical and functional information to enhance diagnostic sensitivity and specificity. Furthermore, radiomics and AI extract high-dimensional features and construct predictive models, enabling precise identification and personalized treatment. This article reviews the progress and clinical applications of these imaging techniques.

## 1 Introduction

BM refers to secondary intracranial malignant tumors formed by hematogenous dissemination of primary cancer cells, and it is also one of the most common complications in patients with malignant tumors. The formation of BM relies on complex interactions between tumor cells and the unique brain microenvironment. Although BM often occur during the advanced stage of cancer, their presence does not necessarily indicate that the disease has reached the terminal stage. As demonstrated in the study by Kamer et al. (1), abnormal expression of specific genes can indirectly promote the occurrence of brain metastasis even in early stage non-small cell lung cancer (NSCLC), and the resulting poor prognosis cannot be ignored.

Medical imaging plays a fundamental role in the diagnosis, staging, and therapeutic response evaluation of BM, which highly depends on techniques with high sensitivity and specificity. However, conventional imaging modalities, including MRI, PET, and CT, all have inherent limitations and are not applicable to all BM. For example, although contrast enhanced MRI is regarded as the gold standard for the diagnosis of BM due to its higher detection rate than contrast enhanced CT (2–4), it shows a significantly increased false positive rate and lower diagnostic efficiency compared with PET in detecting small volume or early stage BM (5). Meanwhile, the detection rate of different types of BM by MRI is also affected by various factors such as contrast agents, magnetic field strength, and scanning sequences.

To overcome the limitations of single imaging modalities, multimodal fusion technologies such as PET/CT have been developed, and emerging technologies including artificial intelligence, radiomics, and radiogenomics have also been applied to improve the clinical detection rate of various types of BM. For instance, Huang et al. (6) combined artificial intelligence with magnetic resonance imaging and established a volume level sensitivity specificity strategy, which reduced the false positive rate of MRI by 44.4% and increased the detection accuracy to 98.7%. Therefore, exploring and optimizing imaging methods is crucial for improving the efficiency of clinical diagnosis and treatment of BM.

MRI, with its exceptional soft-tissue contrast and high sensitivity for small lesions, has become the cornerstone imaging modality for evaluating brain metastases. Accordingly, we begin Section 2 with a comprehensive discussion of MRI, progressing from conventional contrast-enhanced sequences to advanced sequences designed to address specific clinical questions, and we summarize their roles in differential diagnosis (Tables 2, 3). In contrast, CT and PET play adjunctive roles in the routine imaging assessment of brain metastases and are used less frequently; their application is largely confined to scenarios where MRI is contraindicated or unavailable, or where their diagnostic performance has not yet been sufficiently validated. Therefore, CT, PET, and multimodal fusion techniques involving these two modalities are discussed collectively in subsequent sections. These sections explore the ancillary roles of CT and PET, focus on how multimodal fusion (e.g., PET/CT, PET/MRI, as covered in section 3.3) can compensate for the limitations of individual modalities, and offer recommendations for tracer selection based on primary tumor type (section 3.4). In addition, a clinical practice framework is provided in section 4. Through this structure, the review aims to summarize the current state of imaging techniques for brain metastases, highlight their strengths and limitations, and provide a reference for future research and potential clinical applications.

## 2 Applications of MRI in BM

### 2.1 Conventional MRI

Gadolinium-based contrast agents (GBCAs) have been well-validated in contrast-enhanced MRI (2, 7). Because the signal intensity in MRI primarily depends on two intrinsic tissue properties: the T1 relaxation time and the T2 relaxation time. After intravenous injection of a gadolinium-based contrast agent, it distributes throughout the body via the bloodstream, particularly accumulating in tissues with rich blood supply or where the blood-brain barrier is disrupted. The local magnetic field around the gadolinium ions undergoes strong fluctuations. These fluctuations significantly enhance the efficiency with which water protons transfer energy to their surrounding environment. As a result, the T1 relaxation time of nearby water protons is markedly shortened. Although the contrast agent also shortens the T2 relaxation time, at typical clinical doses, the T1-shortening effect predominates.

Current methods to improve the detection rate of BM include:

(1) Increasing magnetic field strength: At 3.0 T, GBCAs generate higher contrast between tumors and normal brain tissue compared to 1.5 T, enabling better detection of BM and leptomeningeal involvement (8).

(2) Increasing gadolinium dose: For gadopiclenol, a dose of 0.1 mmol/kg yields a higher tumor detection rate than 0.08 mmol/kg (9). However, this approach may cause false positives (FPs) and increase patient burden, limiting its widespread clinical use.

(3) In a study by Robert et al. (9), MRI was performed on 18 mice (*n* = 6 per group) to calculate the contrast-to-noise ratio (CNR) between metastatic tumors and healthy parenchyma. The results indicated that gadopiclenol enabled higher diagnostic performance in detecting BM than gadobenate, both at equal (0.1 mmol/kg) and reduced (0.08 mmol/kg) Gd doses, as measured by CNR and the number of lesions detected (2, 10). Moreover, other research corroborates that gadopiclenol at 0.1 mmol/kg is superior to 0.08 mmol/kg for tumor detection, and that both gadoteridol and gadobutrol outperform gadopentetate dimeglumine in detecting metastases.

### 2.2 Hybrid sequences

None of the sequences mentioned in this section are basic sequences like “spin echo” or “gradient echo,” but intelligently combine two separate physical processes (preparation module + high-speed read module).

#### 2.2.1 Sampling perfection with application optimized contrasts using different flip angle evolution (SPACE)

SPACE employs non-selective refocusing pulses and variable flip angles to overcome specific absorption rate (SAR) limitations and T2 decay effects, meeting clinical demands for 3D turbo spin-echo (TSE) imaging. For decades, 2D spin-echo (SE) sequences have been the gold standard for post-contrast imaging of BM. Vymazal et al. (11) compared T1-weighted SPACE and 2D SE in 56 patients with metastatic brain lesions (MBL) showing that T1-weighted SPACE is not inferior to standard thin-slice SE sequences in the detection of MBL.

Prior studies suggest SPACE offers superior diagnostic sensitivity for cerebellar metastases compared to 2D-FLASH (Fast Low-Angle Shot) (12). Additionally, Controlled Aliasing in Parallel Imaging (Wave-CAIPI) post-contrast T1 SPACE provides equivalent lesion conspicuity and diagnostic quality to standard 3D T1 SPACE while reducing scan time by threefold (13). Gule-Monroe et al. (14) compared SPACE and 3D-FLASH in 220 patients, evaluating metastasis count, indeterminate lesions, lesion margins, contrast-to-noise ratio (CNR), artifacts, and overall image quality. Compared with 3D FLASH, the SPACE sequence detected more metastatic lesions and was rated higher for image quality, lesion margin, and CNR, with fewer artifacts. Importantly, the SPACE sequence resulted in increased reader confidence, with fewer indeterminate lesions detected.

#### 2.2.2 Magnetization-prepared rapid gradient-echo (MP-RAGE)

MP-RAGE has been a cornerstone of neuroimaging for decades, providing high-resolution 3D visualization for biopsy guidance, resection planning, and stereotactic radiotherapy. Romano et al. (15) demonstrated its utility in distinguishing glioblastoma (GBM) from metastases. In addition, studies have shown that MP-RAGE is weaker in diagnostic sensitivity for cerebellar metastases than SPACE mentioned above (12).

### 2.3 Advanced MRI black blood imaging technology

Prior to this, the 3D TFE (Turbo Field Echo) sequence had a high signal-to-noise ratio and was widely used to screen BM, and black blood MRI was introduced to distinguish BM from enhanced small blood vessels after contrast injection. Motion-Sensitized Driven Equilibrium (MSDE) is a black-blood MRI technique that suppresses blood signal (unlike bright-blood imaging), enhancing vessel wall visualization and reducing flow-related artifacts (16).

#### 2.3.1 TSE-MSDE imaging

The MSDE-based black blood 3D T1 TSE sequence has achieved a revolutionary improvement in the detection of micrometastases through the strategy of “actively removing interference items (blood signals)” rather than just “passively displaying target items (lesions).” Compared to conventional 3D TFE, MSDE-based black-blood 3D T1 TSE improves small metastasis detection (17). Chen Zhou et al. (18) compared post-contrast 3D T1 GRE (gradient echo) and 3D black-blood sequences in 107 patients, finding that 3D black-blood T1 TSE with MSDE at 1.5T MRI detected more metastases, particularly small lesions, than standard 3D T1 GRE.

#### 2.3.2 iMSDE-TSE imaging

Improved MSDE (iMSDE) adds refocusing pulses and gradients to enhance flow suppression and SNR. Bae et al. (19) evaluated 460 metastases in 30 patients using non-MIP (Maximum Intensity Projection) iMSDE-TSE and non-MIP 3D-GRE. MIP iMSDE-TSE showed comparable figure of merit and sensitivity to non-MIP iMSDE-TSE, both outperforming non-MIP/MIP 3D-GRE (*P* < 0.001). Thus, MIP iMSDE-TSE offers high detectability (equivalent to non-MIP iMSDE-TSE and superior to 3D-GRE) but requires thin-slice correlation to mitigate FPs. Studies also suggest iMSDE-TSE detects more metastases than conventional GRE (C-GRE), aiding equivocal lesion differentiation. (20)

#### 2.3.3 Combined TSE-MSDE and MP-RAGE

Nagao et al. (21) prospectively evaluated 227 patients with suspected metastases using three post-contrast 3D sequences: MPRAGE, TSE-noMSDE, and TSE-MSDE. TSE-MSDE significantly outperformed MPRAGE in FOM (Figure of Merit) (*P* < 0.05) by suppressing vascular signal and improving CNR, albeit with increased FPs. Combining TSE-MSDE/MPRAGE achieved higher FOM than MPRAGE alone while maintaining low FP rates (*P* = 0.004 vs. TSE-MSDE alone). Thus, the hybrid protocol balances diagnostic performance and specificity.

### 2.4 Post-processing techniques in MRI

MIP reflects the X-ray attenuation values of corresponding pixels, enabling visualization of even subtle density variations. It excels in demonstrating vascular stenosis, dilatation, filling defects, and differentiating between vessel wall calcifications and intraluminal contrast agents. When applied to 3D contrast-enhanced VIBE (Volume Interpolated Body Examination) T1-weighted imaging, MIP processing showed no significant difference in sensitivity for detecting small BM (BMs) compared to native images (22). This was also evidenced by a subsequent study by Parillo et al. (22). However, evidence suggests that using MIP-3DT1 reconstructions of previously obtained postcontrast 3D T1W improved detection of BM (23).

### 2.5 Molecular imaging

The tumor-normal brain tissue interface demonstrates elevated endothelial VCAM-1 expression and increased microvascular density (24). Serres et al. (25) developed an iron oxide microparticle-based targeted MRI contrast agent capable of imaging endothelial vascular cell adhesion molecule-1 (VCAM-1). Compared to gadolinium-DTPA-enhanced T1-weighted MRI, T2-weighted MRI following intravenous VCAM-MPIO (microparticles of iron oxide) administration more extensively highlighted the tumor-brain interface in two tumor models. Sites of VCAM-MPIO binding, evident as hypointense signals on MR images, correlated spatially with endothelial VCAM-1 upregulation and bound VCAM-MPIO beads detected histologically (26). Furthermore, Nechaeva et al. showed that RAS70 peptide demonstrated high diagnostic accuracy for GBM and metastases, suggesting potential applications in intraoperative image-guided tumor resection and the development of targeted drug delivery systems (27).

### 2.6 Advanced imaging techniques

In addition to standard MRI protocols, advanced MRI sequences and other imaging techniques can provide lesion-specific information. Although promising for clinical imaging, most of these techniques are still being evaluated in experimental settings and lack standardization.

#### 2.6.1 Chemical exchange saturation transfer MRI (CEST)

The essence of the CEST mechanism is an indirect detection technology based on chemical exchange that “transfers” tiny solute signals and amplifies them to water signals. CEST is sensitive to the exchange of labile protons (including amide protons), rapid exchange of hydroxyl protons, and intramolecular transfer of magnetization from aliphatic (-CH) protons to labile protons, termed relayed nuclear Overhauser effect (rNOE) (28). These protons are found in metabolites such as glutamate, lactate, myo-inositol and glucose, which are vital in a neoplastic milieu (29).

For CEST, both static (30) and dynamic (31) scanning protocols have been employed. In brain tumors, prior studies have demonstrated the potential of glucose as a CEST contrast agent. Wu et al. developed a processing pipeline for *in vivo* glucoCEST (glucose-enhanced CEST) data. To address the low signal-to-noise ratio (SNR) of *in vivo* DGE (dynamic glucose-enhanced) imaging, the pipeline incorporated advanced corrections (dynamic B0 field correction and PCA-based denoising). Its application in metastatic brain tumor patients yielded visually improved DGE maps, enhancing tumor-to-normal tissue DGE contrast in three out of four cases (32).

Amide proton transfer-weighted (APTw) imaging is a subtype of chemical exchange saturation transfer (CEST) imaging and is currently available on 3T MRI systems. Distinguishing brain radionecrosis (RN) from tumor progression (TP) remains a persistent clinical challenge. To address this, Nichelli et al. demonstrated that the fluid-suppressed amide proton transfer-weighted (APTw) metric outperforms leakage-corrected relative cerebral blood volume (rCBV) in differentiating RN from tumor recurrence among patients with evolving SRS (stereotactic radiosurgery)-treated metastases (33). In another context, when assessed within Gd-enhanced tumor areas, the nAPTw (normalized amide proton transfer-weighted) MRI signal intensity—both alone and when combined with DSC (Dynamic susceptibility contrast)-MRI parameters—proved to be an excellent predictor for differentiating GBM from BM (34). However, the small cohort size in this study warrants further investigation.

#### 2.6.2 Magnetic resonance spectroscopy (MRS)

MRS is a non-invasive “biochemical analysis” technique that uses magnetic resonance phenomena to detect the concentration and metabolic state of specific biomolecules in living tissues. It is based on the same physical basis as MRI, but the goal is not to generate anatomical images, but to map a spectrum similar to a “chemical fingerprint.”

MRS can detect metabolic differences between brain tumors, aiding in radiological diagnosis. MRS enables accurate brain tumor characterization at the metabolic level by assessing alterations in key metabolites such as N-acetylaspartate (NAA), choline (Cho), and myo-inositol (mI) (35). AI has further expanded the applications of MRS. For instance, the recently developed Attention Deep-Shallow Network (ADSN) model demonstrated high efficacy in isocitrate dehydrogenase (IDH) mutation detection by extracting relevant features from proton MRS (^1^H-MRS) spectra, achieving an F1-score of 93% in the validation cohort. For telomerase reverse transcriptase gene promoter (TERTp)-only gliomas, the model attained an F1-score of 88% in the validation set, while for TERTp mutation detection, it achieved F1-scores of 80 and 81% in the validation and test sets, respectively (36). The study by Nichelli et al. further underscores the clinical significance of MRS in IDH genotyping. Their findings confirm that edited MRS exhibits high specificity in predicting IDH mutations and facilitates rapid prognostic stratification. With the advent of IDH inhibitor therapies, integrating edited MRS into routine clinical workflows holds considerable promise (37). What’s more, MRS may provide more quantitative, patient-centered, and accurate predictions of treatment response or prognosis (38). (The F1 score is a commonly used metric in machine learning to evaluate the performance of classification models. It can be understood as the weighted average of Precision and Recall. The F1 score is widely applied in machine learning tasks requiring binary or multi-class classification, such as scenarios where diseased samples are far fewer than healthy samples. To ensure a model identifies as many true patients as possible (high recall) while avoiding misclassifying too many healthy individuals as patients (high precision), the F1 score effectively measures this balance.)

MRS is a unique diagnostic tool that utilizes metabolic profiles for brain tumor characterization. Future advancements in MRS should focus on AI and its capability to analyze metabolic data. The integration of AI with MRS represents a significant diagnostic breakthrough, enhancing the accuracy of detecting early tumor progression and differentiating high-grade gliomas from low-grade gliomas.

#### 2.6.3 Diffusion-weighted imaging (DWI)

The principle of DWI is to encode the microscopic random motion of water molecules through a pair of symmetrical gradient pulses, and convert the difference in the diffusion capacity of water molecules in biological tissues into the contrast of the image. DWI is most commonly used to differentiate BM from other intracranial lesions, such as abscesses, because both BMs and abscesses can present as a ring-enhancing lesions on post-contrast T1W imaging. In abscesses, diffusion is usually far more restricted than in BMs, particularly in the central non-enhancing portion (39). By assessing the Brownian motion of water molecules in the microscopic tissue environment and reflecting tissue cellularity through the apparent diffusion coefficient (ADC) values, DWI aids in the characterization of brain tumors and other intracranial pathologies on conventional MR imaging. Early studies suggested that DWI appeared to be the most effective MR imaging technique for distinguishing between two tumor types—hemangioblastomas and BM (40). In recent years, Zhang et al. (41) and He et al. (42) study demonstrated the important value of DWI in distinguishing GBM from high-grade gliomas (HGGs).

#### 2.6.4 Susceptibility-weighted imaging (SWI)

The principle of SWI is to cleverly use the phase information collected by the gradient echo sequence to amplify and convert the small magnetic susceptibility differences in the tissue into extremely vivid image contrast through precise mathematical post-processing. In general, SWI lacks sensitivity in detecting BM (43). Earlier studies demonstrated that SWI may have some diagnostic value for melanoma BM (44). Susceptibility weighted imaging (SWI) is a high-resolution, three dimensional (3D), fully velocity-compensated gradient-echo sequence, which is optimized for magnetic susceptibility effects and more sensitive than conventional MRI sequences for detecting neovascularization or blood products. Additionally, percentage quantification (PQ) of intratumoral susceptibility signals (ITSS) on SWI provides an objective and quantitative method to differentiate certain primary entities of BM. Recently, Sun et al. demonstrated that ITSS PQ aids in the non-invasive characterization of BMs from three lung cancer subtypes, with adenocarcinoma (AD) and non-AD large metastases being distinguishable with high sensitivity and specificity (45).

#### 2.6.5 Perfusion MRI

The principles of advanced imaging techniques are shown in Table 1 below. Perfusion MRI is based on the principle of non-invasively quantifying the blood flow status of tissues by tracing the kinetics of the first passage of a tracer (exogenous contrast agent or endogenous marker blood) in the capillary bed. Since increased tissue perfusion is a hallmark of cancer, perfusion MRI can differentiate BM from normal brain tissue. DSC perfusion MRI is the most commonly used technique for measuring rCBV. DCE and ASL are less frequently employed. Additionally, research by Su Y. demonstrated that analyzing diffusion, perfusion, and structural features within the enhanced tumor area (ETA) can effectively differentiate high-grade glioma (HGG) from solitary BM (46).

**TABLE 1:** Principles underlying advanced imaging techniques.

Technique	Biomarker	Primary clinical application	Pitfalls	Advantages
Diffusion-weighted imaging (DWI)	Apparent diffusion coefficient	Acute cerebral infarction (core application), tumor characterization,	Susceptibility artifacts at skull base, non-specificity	Fast, gold standard for stroke
Diffusion tensor imaging (DTI)	Fractional anisotropy, mean diffusivity	White matter fiber tractography and mapping	Fiber crossing issues	Pre-surgical planning, visualizes tracts
Dynamic susceptibility contrast (DSC)	Relative cerebral blood volume mean transient time	Assessment of cerebral perfusion	Contrast agent leakage causing quantification errors	Tumor, grading, differentiates recurrence/necrosis
Dynamic contrast-enhanced (DCE)	Contrast transfer coefficient (Ktrans)	Assessment of vascular permeability	Highly dependent on pharmacokinetic models	Quantifies vascular leakage
Arterial spin labeling (ASL)	Cerebral blood flow (CBF)	Quantitative measurement of CBF	Low signal-to-noise ratio	Completely non-invasive (no need for contrast injection)

A summary of this part of the study is shown in Table 2.

**TABLE 2:** Advanced imaging techniques.

Technology	CEST	MRS	DWI	SWI	Perfusion MRI
Authors	Wu et al. (32)	Sacli-Bilmez et al. (55)_	She et al. (40)	Sun et al. (45)	Knutsson et al. (34)
Number of patients	4	225	51	213	18
Conclusion (Clinical application prospects)	A direct comparison of DGE CEST and ^18^F-fluorodeoxy-D-glucose PET was achieved in patients with BM.	Deep learning models accurately predicted the IDH and TERTp mutational subgroups of hemispheric diffuse gliomas	DWI and DSC-PWI are helpful in the characterization and differentiation of hemangioblastomas from BM.	PQ of ITSS can contribute to the non-invasive characterization of BMs from the three subtypes of lung cancer.	nAPTw MRI signal intensity alone or combined with DSC-MRI parameters, was an excellent predictor for differentiating GBM and BM.
Limitations	Dynamic AIF (arterial input function) detection not achieved; optimal component number requires validation.	Single-center; short-TE MRS limited for small/overlapping metabolite detection.	Retrospective; few non-lung primary metastases included.	Small macrometastasis sample; large cell/carcinoid subtypes not included.	Small cohort; heterogeneous BM group (lung adenocarcinoma/melanoma).

### 2.7 The utility of the aforementioned techniques in the radiological detection of BM

These advanced MRI techniques provide a certain degree of improvement in the diagnosis of BM. For instance, SPACE and TSE-MSDE imaging can increase the detection rate of small metastases. In addition, many techniques play an important role in differential diagnosis (Table 3).

**TABLE 3:** Applications of advanced imaging techniques in differential diagnosis.

Types of lesions to differentiate from BM	Recommended MRI technique(s)	Key discriminating imaging features	Reported diagnostic performance (Reference)	Limitations/ comments
Abscess	DWI	Restricted Diffusion: Markedly hyperintense on DWI, low ADC values in the central cavity. BM: Usually facilitated diffusion in necrotic core.	Sensitivity/Specificity: > 95% in typical pyogenic abscesses (39).	False negatives possible in fungal or tubercular abscesses.
Subtypes of Lung Cancer BM	SWI	ITSS Percentage: Higher ITSS PQ in Small Cell Lung Cancer (SCLC) and Squamous Cell Carcinoma vs. Adenocarcinoma.	ITSS PQ Sensitivity: 77.8%, Specificity: 82.4% for non-adenocarcinoma vs. adenocarcinoma (45).	Limited data on large cell carcinoma or carcinoid tumors.
IDH and TERTp mutations in diffuse gliomas	MRS	Metabolic Profile: 2-Hydroxyglutarate (2-HG) peak detection for IDH mutation.	Deep Learning Model (ADSN): F1-score of 93% for IDH, 81% for TERTp (36)	Short-TE MRS may have limited sensitivity for overlapping metabolites.
Glioblastoma (GBM)	MP-RAGE/nAPTw MRI signal intensity alone or combined with DSC-MRI parameters/DWI	APTw: Lower signal intensity in GBM compared to BM.	APTw + DSC: Excellent predictor for differentiation (34). VOI (volume of interest)-based DWI/MP-RAGE: AUC > 0.80 (15).	Perfusion parameters may overlap if BM is highly vascular (e.g., melanoma, renal).

## 3 Other imaging techniques

### 3.1 CT

CT features rapid imaging acquisition and serves as the first-line imaging modality for initial intracranial evaluation and identification of potential neurosurgical emergencies in the emergency setting. It exhibits distinct advantages in depicting acute hemorrhage, calcification and bony structures, and has been demonstrated to possess significantly higher sensitivity than RN scanning for the detection of these abnormalities. Its inherent low soft-tissue resolution renders it difficult to reliably detect isodense metastases with density similar to that of normal brain parenchyma. In addition, the associated ionizing radiation exposure during examination constitutes a relevant concern. More importantly, CT demonstrates low sensitivity for the detection of small-volume BM.

In comparison, MRI affords far superior soft-tissue contrast resolution relative to CT and delineates the anatomical details of brain structures with high spatial precision. Accordingly, MRI (commonly with contrast enhancement) is widely regarded as a more reliable tool for the accurate diagnosis of BM in clinical practice. This diagnostic discrepancy has been quantitatively validated in studies: the detection rate was significantly elevated from approximately 10 to 24% when MRI was adopted instead of CT for BM screening (3). This marked increment (14%) is mainly ascribed to the limited capability of CT in identifying micrometastases.

Notably, the rise of AI has provided a novel strategy for enhancing the application value of CT in the diagnosis and treatment of BM. AI enables the extraction of deep features from CT images that are imperceptible to the human eye, which can partially compensate for the deficiencies of conventional CT interpretation. To verify this potential, Cha et al. (47) developed a convolutional neural network and applied it to predict the response of patients with BM to SRS. The model achieved favorable predictive accuracy (area under the curve, AUC = 0.86), strongly supporting the feasibility of integrating CT-based AI radiomics into clinical decision-making for the management of BM.

### 3.2 PET

PET demonstrates significant advantages in early diagnosis of BM due to its unique functional imaging characteristics. Compared to conventional imaging techniques (MRI, CT) (Table 4). PET provides superior specificity in differentiating treatment-related changes from residual or recurrent disease through metabolic imaging mechanisms using radiotracers (e.g., ^18^F-FDG) (48, 49), particularly showing outstanding detection efficacy for small-volume or early-stage brain metastatic lesions (5). In clinical practice, the most widely used PET tracer is the glucose analog ^18^F-FDG, which serves as the primary tool for whole-body assessment during initial diagnosis, demonstrating high sensitivity for screening multiple metastases and larger lesions. However, its detection capability for micro-metastases or low-metabolic-activity BM is limited by the low signal-to-noise ratio between tumor and normal brain tissue (i.e., tumor-to-background ratio, TBR) (48, 50, 51). To reduce physiological ^18^F-FDG uptake in normal brain tissue, current protocols require at least 6 hs of fasting prior to scanning, placement of subjects in a quiet environment with eye masks during the tracer uptake phase, and implementation of delayed imaging strategies (50). Furthermore, ^1^ 8 F-FDG PET has been proven unsuitable for radiotherapy target delineation due to limited spatial coverage of hypermetabolic areas. Its low specificity in single-modality assessment for post-radiotherapy evaluation further diminishes its value in treatment follow-up studies for BM (50). Consequently, multimodal imaging fusion techniques such as ^18^F-FDG PET/CT and ^18^F-FDG PET/MRI are routinely employed to significantly enhance comprehensive clinical research and application value.

**TABLE 4:** Performance comparison of PET, CT, and MRI for diagnosing BM.

Dimension	PET	CT	MRI
Core advantages	Provides metabolic information; high detection rate for small/early-stage BM; suitable for staging evaluation	Fast scanning speed; sensitive to calcification, hemorrhage, and skull involvement; enables rapid assessment of intracranial emergencies	High soft-tissue resolution; clearly delineates lesion morphology and boundaries; gold standard for BM diagnosis
Diagnostic performance	Detection rate:approximately 24.7% [*n* = 393(30)]	Detection rate:approximately 10% [*n* = 481(23)]	Detection rate:approximately 76.7% [*n* = 393(30)]
Main limitations	Low spatial resolution; high radiation dose; limited specificity (false positives common in inflammation)	Low soft-tissue resolution; insufficient detection of microlesions (< 5 mm often missed); radiation exposure	Long scanning time; contraindicated in patients with metallic implants (e.g., pacemakers, aneurysm clips); claustrophobia
Level of evidence (QUADAS-2)	Moderate (retrospective)	Low to moderate (retrospective)	Moderate (retrospective)

Available diagnostic performance data across references are limited. Overall, MRI demonstrates the best efficacy for detecting BM, followed by PET, with CT being the most limited.

Compared to ^18^F-FDG, amino acid PET tracers such as ^11^C-methionine (^11^C-MET), ^18^F-fluoroethyltyrosine (^11^C-MET), ^18^F-fluoroethyltyrosine (^18^F-FET), and ^18^F-fluorodopa (^1^8 F-FDOPA) are frequently used as brain-specific complementary diagnostic tools for BM in biopsy localization, surgical resection planning, and post-radiotherapy assessment, owing to their higher cerebral signal-to-noise ratios and uptake mechanisms independent of blood-brain barrier (BBB) disruption (52). These tracers target the L- γ-type amino acid transporters (LAT) overexpressed in BM, leading to characteristic high uptake of amino acids like ^1^ 8 F-FET in metastatic lesions, thereby enabling effective lesion information capture (52). Typical manifestations include: in ^1^ 8 F-FET PET imaging, radiation necrosis areas usually exhibit a progressively increasing time-activity curve pattern, whereas recurrent metastatic lesions predominantly demonstrate early peak uptake followed by plateau or continuous decline kinetics (48). However, the short physical half-life (20.4 min) of carbon-11 in ^11^C-MET significantly limits its clinical application (49). A comparative study demonstrated that the accuracy of MRI and ^18^F-FET PET for identifying treatment-related changes in non-responders was 60 and 80%, respectively, suggesting that amino acid PET is superior to MRI in distinguishing treatment-related changes from tumor recurrence (53).

Beyond these tracers, researchers have identified various novel targeted PET tracers: Prostate-Specific Membrane Antigen (PSMA) imaging demonstrates 27% superior diagnostic accuracy compared to conventional imaging, directly leading to treatment regimen modifications in 28% of cases (54). Fibroblast Activation Protein Inhibitor (FAPI) has been proven in PET/CT imaging to outperform ^18^F-FDG in detecting brain tumors. In the field of theranostics, diagnostic-therapeutic PET tracers can molecularly conjugate diagnostic targeting ligands with PET radionuclides within the framework of pharmaceutical and radiopharmaceutical development, thereby establishing PET-based systems for targeted radionuclidetherapy. Such tracers show promise as non-invasive whole-body visualization navigation tools and may serve as predictive biomarkers through molecular imaging characteristics to precisely identify patient populations most likely to benefit from targeted therapies (50).

### 3.3 Multimodal fusion technologies

A single imaging modality can no longer meet the clinical demand for precise detection of BM, leading to the development of a multimodal fusion technology system that integrates multi-source medical imaging data to optimize BM diagnosis, localization, and treatment planning. Fusion technologies such as PET CT and PET/MRI exhibit differential performance characteristics in key aspects of BM detection and treatment response assessment due to the functional properties and clinical application differences of their constituent modules.

#### 3.3.1 PET/CT

The core value of PET/CT fusion technology stems from the deep complementarity of its multimodal information: PET quantitatively characterizes tumor metabolic activity using radiotracers (e.g., ^18^F-FDG), while CT simultaneously provides high-resolution anatomical structural information, enabling precise spatial localization of tumors and their relationship with adjacent tissues. This synergistic integration of functional metabolism and anatomical structure significantly enhances the sensitivity of PET/CT in the initial differential diagnosis and treatment staging of BM. Empirical studies confirm that combined analysis of dynamic and static ^18^F-fluoroethyltyrosine (^18^F-FET) PET/CT parameters achieves optimal diagnostic performance in differentiating RN from tumor recurrence (TR) after stereotactic radiosurgery, with dynamic PET offering additional clinical benefits by potentially avoiding unnecessary biopsies (55).

However, radiolabeled amino acids such as ^18^F-FET are costly and not widely available compared to ^18^F-fluorodeoxyglucose (^18^F-FDG), and they have not yet received (Food and Drug Administration) FDA approval. This has driven research toward optimized ^18^F-FDG PET/CT protocols, such as dual-phase imaging and delayed-phase imaging. These strategies leverage the kinetic properties of ^18^F-FDG, which shows persistent uptake and accumulation in tumor tissue over time but gradual washout in normal brain tissue and inflammatory lesions, thereby overcoming the technical limitation of low TBR. A study using a short-interval dual-phase ^18^F-FDG PET/CT protocol (acquisition time points: 30 and 90 min post-injection) to differentiate TR from RN demonstrated excellent performance with 95% sensitivity, 91% specificity, and 93% diagnostic accuracy while ensuring patient tolerance and scanner throughput efficiency (56).

Beyond conventional ^18^F-FDG PET/CT, targeted receptor PET/CT techniques for different molecular targets show potential for superior sensitivity in BM detection. For example, 68Ga-Trivehexin, a tracer targeting integrin αvβ6—a biomarker highly expressed on various malignant tumor cells—unexpectedly visualized BM from squamous cell carcinoma (SCC) in a study initially designed for SCC detection (57). Cheng et al. (51) applied ^18^F-BCH PET/CT and not only identified known lesions but also detected two additional small metastases missed by concurrent ^18^F-FDG PET/CT, later confirmed by brain MRI. In rare cases, PET/CT has even outperformed MRI in sensitivity; Wang et al. (58) reported a clinical case where PET/CT detected an abnormal neuroblastoma BM in the left cerebellopontine angle that was missed by MRI. Laura et al. (59) noted that while MRI had a high specificity (97.6%) for detecting prostate cancer CNS (central nervous system) metastases, its sensitivity was relatively low (59.4%), whereas 68Ga-PSMA PET/CT achieved a pooled sensitivity of 98%. Despite these exceptional cases of high sensitivity, PET/CT is generally not the first-line screening or diagnostic tool for BM in routine clinical practice. Brain MRI remains significantly superior to PET/CT in BM detection. Data clearly show that the estimated cumulative detection rates for BM over 5 years were 24.7% for PET/CT and 76.7% for MRI (60), firmly establishing MRI’s dominance in this field.

#### 3.3.2 Multiparametric MRI (mpMRI)

Fusion technology integrates data from multiple sequences, including T1-weighted imaging (T1WI), T2-weighted imaging (T2WI), diffusion-weighted imaging (DWI), perfusion-weighted imaging (PWI), and magnetic resonance spectroscopy (MRS), to comprehensively analyze the morphological structure, metabolic information, and tumor microenvironment characteristics of BM. This approach aims to enhance the accuracy of diagnosis and therapeutic evaluation for BM. In recent years, radiomics models based on mpMRI have become a research hotspot in this field. By incorporating AI techniques, these models enable effective differentiation, diagnosis, and prediction of disease status and treatment response. For instance, Zhang et al. (61) developed an mpMRI-based radiomics model to distinguish BM originating from lung adenocarcinoma versus non-lung adenocarcinoma, demonstrating high predictive accuracy (AUC = 0.824). Peng et al. (47) experimentally validated the utility of an MRI radiomics model in differentiating radiation-induced brain injury from true tumor progression. Their final predictive model achieved an AUC of 0.81, a specificity of 65%, and a sensitivity of 87%. In contrast, radiologists were only able to accurately classify 73% of cases, with a sensitivity of 97% and a specificity of 19%.

In another study, Kniep et al. (47) aimed to predict the primary origin of BM, including cases from small-cell lung cancer (SCLC), NSCLC, gastrointestinal cancer, melanoma, and breast cancer. The model’s predictions were compared with traditional radiological assessments by two radiologists. The final model achieved an AUC of 0.64 for NSCLC-derived BM and 0.82 for melanoma-derived BM, with overall predictive performance surpassing that of conventional radiological interpretation.

Looking ahead, with the advancement of 7T ultra-high-field MRI technology and sophisticated AI algorithms, mpMRI fusion is expected to play an increasingly vital role in the early diagnosis and precision treatment of BM.

#### 3.3.3 PET/MRI

It integrates the molecular metabolic information of PET with the high-resolution soft-tissue imaging capability of MRI, enabling the precise identification of BM from both functional and anatomical perspectives. Compared with PET/CT, its advantages are mainly reflected in three aspects: no ionizing radiation, excellent soft-tissue contrast, and more accurate registration of anatomical and functional metabolic images—all of which significantly improve the overall quality of scanned images. Compared with MRI alone, PET/MRI overcomes the limitations of traditional MRI in evaluating the progression of disease (POD) and treatment-related changes (TRC) in patients with brain tumors. In addition, it can effectively utilize the inherent multi-parameter imaging capability of MRI to improve diagnostic accuracy, thereby reducing false-negative results during detection. In a study involving 42 patients with central nervous system gliomas and 38 patients with BM from different primary sites, 18F-FET PET/MRI was used for efficacy evaluation. The results showed that the diagnostic accuracy of this technique in patients with BM reached 95%, which was significantly higher than that of PWI alone (76%) or traditional MRI (66%) (62). This effectively reduced the misdiagnosis rate to a certain extent.

PET/MRI can also be combined with radiomics methods to optimize diagnostic performance by extracting quantitative features, precisely locating target areas, and obtaining data such as metabolic heterogeneity and quantitative imaging characteristics from PET/MRI multimodal images. Lohmann et al. (47) performed 18F-FET PET/MRI scans on patients with abnormal BM detected by MRI. The results showed that 18F-FET PET/MRI combined with radiomics achieved a diagnostic accuracy of 89% (specificity 96%, sensitivity 85%). Compared with conventional MRI, this combined strategy shows a significant advantage in achieving higher specificity.

Furthermore, the type of PET tracer selected has a significant impact on the detection results. Studies have confirmed that compared with 18F-FDG PET/MRI or PET/MRI using other amino acid (AA) tracers, 18F-FACBC PET/MRI exhibits a higher TBR. It can even detect tiny lesions located in the cerebellum (63). However, PET/MRI still has limitations, such as excessively long scanning times, relatively high costs, and a lack of unified standards for image analysis and interpretation. These factors have led to its low clinical penetration. More prospective studies are needed in the future to fully validate its clinical application value.

### 3.4 Clinical applications of the above techniques

#### 3.4.1 Optimizing tracer selection according to primary tumor type

Brain metastases from prostate cancer: SMA-targeted PET/CT or PET/MRI is preferred, as it exhibits higher sensitivity than ^18^F-FDG.

Neuroendocrine tumors or certain adenocarcinomas :68Ga-labeled tracers (e.g., 68Ga-DOTATATE) are recommended, which can detect small metastases negative on MRI in some cases.

Other solid tumors (lung cancer, breast cancer, melanoma, etc.): ^18^F-FDG PET/CT remains the routine clinical choice; however, false negatives may occur with some hypometabolic or small lesions. Dual-phase or delayed imaging is advised to improve the TBR.

When differentiation between recurrence and RN is challenging: Dynamic PET or PET/MRI combined with ^18^F-FDG is the first-line approach to avoid unnecessary biopsies, regardless of the primary tumor type.

## 4 Clinical imaging framework for brain metastases

Based on the reviewed literature, we propose a structured clinical framework (Table 5) to guide the choice of imaging techniques according to common clinical scenarios in patients with suspected or confirmed BM. The table below summarizes the recommended first-line and adjunctive imaging modalities, their key diagnostic role.

**TABLE 5:** Opinions on imaging detection of BM.

Clinical scenario	First-line imaging	Adjunctive/ problem-solving techniques	Key diagnostic goal
Screening for BM in high-risk cancer patients (asymptomatic)	Contrast-enhanced MRI (3D T1-weighted, e.g., MP-RAGE or SPACE)	–	Detect small (< 5 mm) and early BM
Suspected BM on CT or MRI (confirmation and characterization)	Contrast-enhanced MRI (including DWI, SWI, perfusion)	PET/CT with amino acid tracer (^18^F-FET or ^11^C-MET) if MRI equivocal	Differentiate BM from other ring-enhancing lesions (abscess, primary brain tumor)
Differentiation of solitary BM from glioblastoma (GBM)	MRI with DWI, DSC-perfusion, and APTw (if available)	^18^F-FET PET/MRI or radiomics model	Preoperative differential diagnosis
Distinguishing tumor recurrence from radiation necrosis after SRS	Dynamic or dual-phase ^18^F-FDG PET/CT, or ^18^F-FET PET	Perfusion MRI (DSC, DCE) and APTw MRI	Avoid unnecessary biopsy, guide treatment
Detection of BM in patients with MRI contraindications (e.g., pacemaker, claustrophobia)	Contrast-enhanced CT (with thin slices)	PET/CT (^18^F-FDG or amino acid)	Emergency assessment and screening

The framework is intended as a practical reference for clinicians and radiologists. In daily practice, contrast-enhanced MRI remains the cornerstone for most scenarios. Advanced MRI techniques (DWI, SWI, perfusion, APTw) and PET (especially with amino acid tracers) should be considered when conventional MRI is inconclusive or when specific clinical questions arise (e.g., recurrence vs. necrosis, differentiation from GBM). For resource-limited settings, contrast-enhanced CT may be used as a screening alternative, albeit with lower sensitivity. Emerging techniques such as radiomics, radiogenomics, and liquid biopsy are not yet ready for routine clinical use but are promising for future personalized approaches.

## 5 Future perspectives

### 5.1 Radiomics

It involves the high-throughput extraction of a large number of quantitative features—such as shape, intensity, texture, and wavelet features—that are imperceptible to the human eye from routine medical images (e.g., T1-weighted, T2-weighted, DWI, DCE, SWI sequences from MRI). These features are analyzed using machine learning or AI methods to construct models for diagnosis, prognostic prediction, or treatment response assessment. The application of deep learning is equally extensive. Radiomics is also frequently employed to differentiate metastases from other intracranial lesions. As a common malignant brain tumor, BM often exhibit imaging characteristics that overlap with those of GBM, posing a considerable diagnostic challenge for clinicians. Studies have shown that deep learning models applied to conventional MRI can aid in preoperatively distinguishing GBM from solitary BM, achieving diagnostic accuracy superior to that of neuroradiologists (64). Multi-sequence radiomic models represent an effective method for differentiating hemorrhagic brain metastasis covered by hematoma (HBM.cbh) from non-neoplastic intracranial hematomas (nn-ICH). When combined with clinical and radiological features, they provide the optimal predictive model for diagnostic performance (65). Some scholars use T1-enhanced BM MRI to distinguish between lung squamous cell carcinoma and adenocarcinoma through radiology-based machine learning (66). AI-based multimodal fusion technology is also evolving, and Linyan et al. (67) proposed a novel multimodal fusion network called MFN-VAE, MFN-VAE effectively distinguishes between SCLC and NSCLC with high accuracy (AUC: 0.920) (67).

### 5.2 Radiogenomics

**FIGURE 1:**
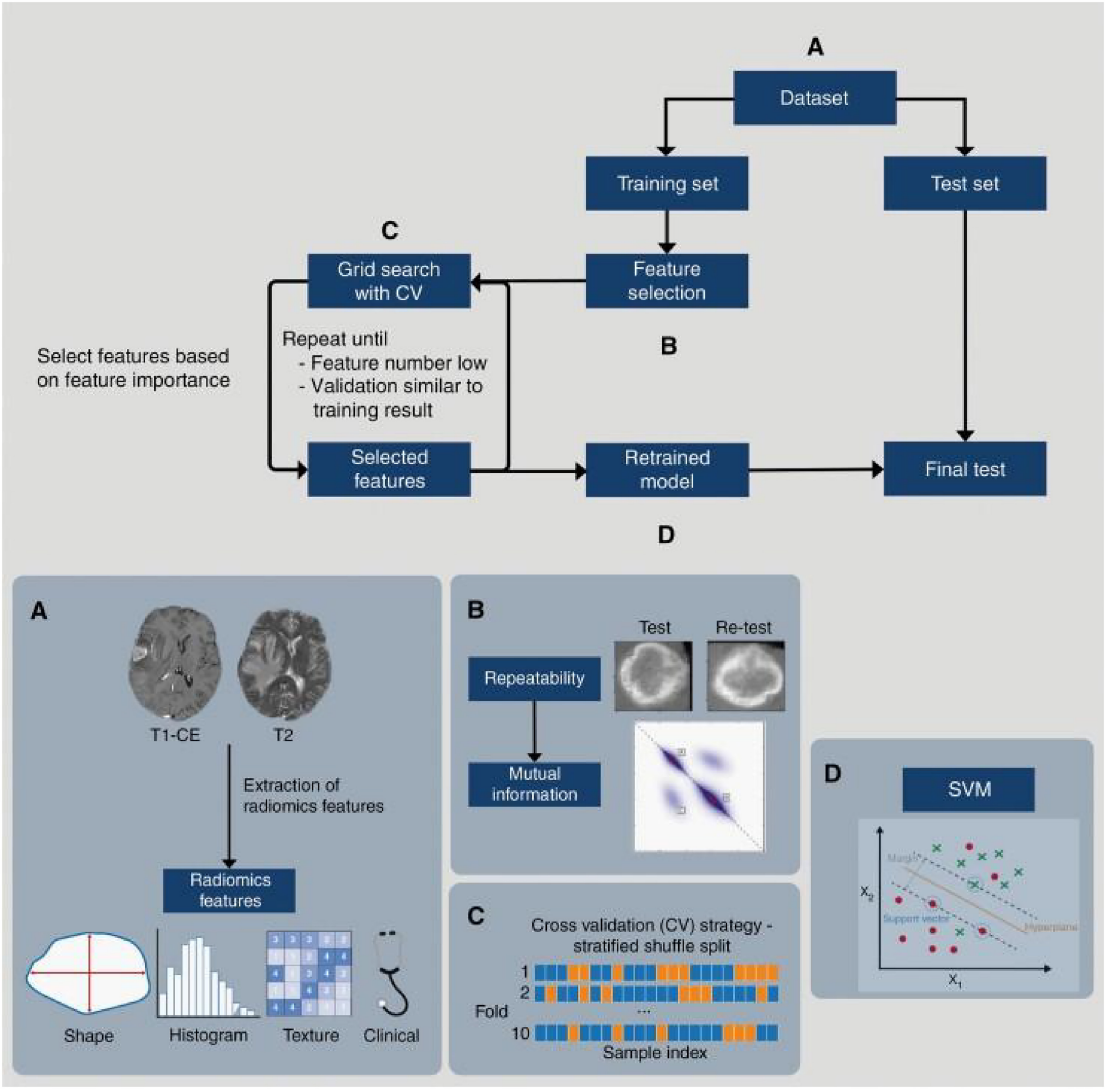
Radiomics workflow. **(A)** The dataset was divided in a training set (University Hospital Cologne) and test set (University Hospital Regensburg). For both, radiomics features were extracted. **(B)** Repeatability was assessed for features calculated on the original image and an augmented version of the image. The 100 radiomics features with highest mutual information were selected from the subset of repeatable features. **(C)** Ten-fold stratified shuffle split cross validation was performed until the validation accuracy did not further improve. **(D)** Using the optimal model parameters and best performing features, the model was retrained on the complete training data. In a last step the model was applied to the test set. CV, cross validation; SVM, support vector machine; T1-CE, contrast-enhanced T1-weighted MRI; T2, T2-weighted MRI.

This is an advanced branch of radiomics. It aims to establish associations between radiomic features extracted from images and the underlying gene expression, molecular subtypes, or gene mutation status of tumors. Its core objective is to infer the molecular characteristics of a tumor through a “non-invasive biopsy.” For example, Meißner AK et al. developed a machine learning model based on clinical parameters and MRI radiomic features (Figure 1) that can predict the intracranial BRAF V600E mutation status in melanoma BM with high diagnostic accuracy (AUC 0.92), thereby providing guidance for the subsequent use of BRAF inhibitors (68). Deng constructed an interpretable prognostic model to predict overall survival in patients with BM from NSCLC by analyzing extracted radiological features from T1/T2 MRI scans and integrated RNA sequencing data to personaliz treatment strategies and improved clinical outcomes (69).

### 5.3 Liquid biopsy

Iquid biopsy is a non-invasive diagnostic technique that identifies signs of disease, particularly cancer, by analyzing samples of body fluids. While blood is the most commonly used sample, other fluids such as urine, cerebrospinal fluid (CSF), and saliva can also be utilized. However, current methods have limited efficacy in diagnosing BM due to biomarker instability and low detectable levels of tumor-specific biomarkers. To address this issue, Premachandran et al. (70) integrated liquid biopsy with the aforementioned radiogenomics. By utilizing extracellular vesicles (EVs) as biomarkers for BM and employing a brain nanosensor, they established a machine learning model for metastatic tumors *in vitro*. This model achieved 97% sensitivity in distinguishing BM from primary brain tumors and 100% accuracy in predicting lung metastasis (70). Similarly, Zuccato et al. (71) used methylomes from cerebrospinal fluid (CSF) to brain tumors to develop Binomial GLMnet classifiers, which also demonstrated significant progress in the differential diagnosis of BM, achieving an area under the receiver operating characteristic curve (AUROC) of 0.93 (95% CI: 0.71–1.0) for BM, 0.83 (95% CI: 0.63–1.0) for GBM, and 0.91 (95% CI: 0.66–1.0) for central nervous system lymphoma (CNSL) (71).

### 5.4 Quantitative sensitivity mapping (QSM)

QSM is a relatively new MRI technique used to quantitatively image the spatial distribution of tissue magnetization properties. QSM helps detect traumatic brain injury, cerebral microhemorrhage, by analyzing changes in substances such as iron and calcium and measuring the magnetic susceptibility of tissues. As early as 2016, studies have demonstrated the role of QSM in BM, especially in the internal capsule and cerebrospinal fluid (72). However, the use of high-resolution QSM can reduce SNR, which can affect the quality of diagnosis. Doniza et al. (73) used Marchenko-Pastur Principal Component Analysis (MP-PCA) to denoise T2*weighted (T2*w) data, which improved the quality of QSM maps and was validated in patients with BM (73).

## 6 Discussion

### 6.1 Summary of key findings

This narrative review of 78 articles provides a comprehensive overview of current imaging techniques for BM. Several consistent findings emerge. MRI remains the diagnostic cornerstone. Conventional contrast-enhanced MRI, particularly 3D T1-weighted sequences such as MP-RAGE and SPACE, offers the high sensitivity for detecting BM, including small (< 5 mm) lesions. Advanced MRI sequences further improve diagnostic performance in specific scenarios: SPACE and black-blood TSE-MSDE increase the detection rate of small metastases and reduce indeterminate findings; APTw imaging and perfusion MRI (DSC, DCE) help differentiate BM from glioblastoma and radiation necrosis; DWI reliably distinguishes BM from abscesses; and SWI with ITSS quantification aids in characterizing BM from lung cancer subtypes.

PET adds value when MRI is inconclusive. Amino acid PET tracers (^18^F-FET, ^11^C-MET) exhibit higher tumor-to-background ratios than ^18^F-FDG and are superior for differentiating recurrent tumor from radiation necrosis after stereotactic radiosurgery. Dynamic or dual-phase ^18^F-FDG PET/CT protocols also improve specificity in this setting. However, the limited availability and high cost of amino acid tracers restrict their routine use.

Multimodal fusion and AI are promising but not yet mature. PET/CT, PET/MRI, and mpMRI integrate complementary information and can enhance diagnostic accuracy. Radiomics and deep learning models show potential for predicting molecular subtypes (e.g., BRAF mutation, IDH status) and distinguishing primary origins of BM, but most models lack external validation and standardized implementation.

The present review offers three novel contributions. First, it systematically compares the diagnostic performance of emerging MRI sequences (SPACE, black-blood TSE-MSDE, APTw, ITSS) against standard protocols, highlighting incremental benefits and trade-offs (e.g., increased false positives with TSE-MSDE). Second, it includes the most recent literature (2024–2025) on AI-based radiomics and liquid biopsy. Third, Table 5 provides a clinically actionable framework that summarizes first-line and problem-solving imaging choices for common clinical dilemmas.

### 6.2 Limitations

Furthermore, this article possesses certain limitations. Most of the original studies cited in this review were retrospective in design with relatively small sample sizes. This limitation restricts the generalizability of the reported diagnostic performance metrics, including sensitivity, specificity, and area under the curve. For the majority of advanced imaging techniques addressed in this review, prospective and multi-center validation studies are still lacking. This review focuses primarily on high-end and cutting-edge imaging modalities and highlights their clinical value in the detection of small BM as well as in the differential diagnosis of BM. However, we acknowledge that such imaging modalities are not widely available in many resource-limited or primary care settings. Consequently, this review does not provide practical guidance for optimizing brain metastasis screening strategies under these real-world constraints, representing an important knowledge gap to be addressed in future research.

### 6.3 Future research directions

To advance the field, future research should prioritize the following areas.

Prospective, multi-center validation—Most advanced MRI and PET findings come from single-center studies. Multi-center prospective trials using standardized imaging protocols are urgently needed to confirm diagnostic performance and define thresholds.

Standardization of AI/radiomics—Radiomics and deep learning models should undergo external validation on independent datasets, and open-source code/features should be shared to enable reproducibility.

Resource-adapted strategies—Given that high-end imaging is unavailable worldwide, research should also explore low-cost alternatives (e.g., AI-assisted CT, abbreviated MRI protocols) for screening BM in primary or resource-limited settings.

## 7 Conclusion

In summary, MRI remains the cornerstone for the current diagnosis and prognostic evaluation of BM, with each of its diverse imaging modalities offering unique advantages (such as those previously mentioned for screening small metastases and differential diagnosis). However, given the complexity and diversity of imaging techniques, identifying the optimal sequence combinations is crucial for accurate diagnosis. When differentiating BM from other intracranial lesions or treatment-related effects, more advanced imaging modalities (PET and multimodal MRI sequence fusion) may provide clinically valuable information. Current research indicates that radiomics and radiogenomics are rapidly evolving in optimizing BM detection. Ultimately, these research efforts aim to enhance the accuracy and sensitivity of BM imaging detection, particularly for small metastases.

## 8 Materials and methods

In this narrative review, we analyzed the latest advances in imaging detection of brain metastases and highlighted future perspectives in this field. A literature search was performed in PubMed using combinations of the following terms: “Brain Metastases” and “Imaging Detection.” Studies were restricted to articles published between 1984 and 2025 (predominantly 2020–2025). A total of 9,839 papers were identified, of which 78 were included (All 78 articles are cited), encompassing clinical studies, clinical trials, and other relevant publications. Potentially eligible publications were identified by one author and confirmed by consensus. As this is a narrative review, we did not perform a formal quality scoring (e.g., QUADAS-2). But, we descriptively assessed each study for methodological features (prospective vs. retrospective design, sample size, blinding, reference standard) and discuss these limitations in the text and in Tables 2, 3.

## Data Availability

The original contributions presented in this study are included in the article/supplementary material, further inquiries can be directed to the corresponding authors.

## References

[1] Kamer I Steuerman Y Daniel-Meshulam I Perry G Izraeli S Perelman M et al. Predicting brain metastasis in early stage non-small cell lung cancer patients by gene expression profiling. *Transl Lung Cancer Res*. (2020) 9:682–92. 10.21037/tlcr-19-477 32676330 PMC7354143

[2] Kuhn MJ Hammer GM Swenson LC Youssef HT Gleason TJ. MRI evaluation of “solitary” brain metastases with triple-dose gadoteridol: comparison with contrast-enhanced CT and conventional-dose gadopentetate dimeglumine MRI studies in the same patients. *Comput Med Imaging Graph*. (1994) 18:391–9. 10.1016/0895-6111(94)90011-6 7954317

[3] Seute T Leffers P ten Velde GP Twijnstra A. Detection of brain metastases from small cell lung cancer: consequences of changing imaging techniques (CT versus MRI). *Cancer*. (2008) 112:1827–34. 10.1002/cncr.23361 18311784

[4] Suzuki K Yamamoto M Hasegawa Y Ando M Shima K Sako C et al. Magnetic resonance imaging and computed tomography in the diagnoses of brain metastases of lung cancer. *Lung Cancer*. (2004) 46:357–60. 10.1016/j.lungcan.2004.05.011 15541821

[5] Suh JH Kotecha R Chao ST Ahluwalia MS Sahgal A Chang EL. Current approaches to the management of BM. *Nat. Rev. Clin. Oncol.* (2020) 17:279–99. 10.1038/s41571-019-0320-3 32080373

[6] Huang Y Bert C Sommer P Frey B Gaipl U Distel LV et al. Deep learning for BM detection and segmentation in longitudinal MRI data. *Med Phys.* (2022) 49:5773–86. 10.1002/mp.15863 35833351

[7] Russell EJ Geremia GK Johnson CE Huckman MS Ramsey RG Washburn-Bleck J et al. Multiple cerebral metastases: detectability with Gd-DTPA-enhanced MR imaging. *Radiology*. (1987) 165:609–17. 10.1148/radiology.165.3.3317495 3317495

[8] Ba-Ssalamah A Nöbauer-Huhmann IM Pinker K Schibany N Prokesch R Mehrain S et al. Effect of contrast dose and field strength in the magnetic resonance detection of BM. *Invest Radiol.* (2003) 38:415–22. 10.1097/01.RLI.0000067488.57101.bd 12821855

[9] Robert P Vives V Rasschaert M Hao J Soares M Lemaître M et al. Detection of brain metastases by contrast-enhanced MRI: comparison of gadopiclenol and gadobenate in a mouse model. *Invest Radiol*. (2024) 59:131–9. 10.1097/RLI.0000000000001032 37921777 PMC11441733

[10] Fan B Li M Wang X Xu Y Li F Zhang L et al. Diagnostic value of gadobutrol versus gadopentetate dimeglumine in enhanced MRI of brain metastases. *J Magn Reson Imaging*. (2017) 45:1827–34. 10.1002/jmri.25491 27696616

[11] Vymazal J Ryznarova Z Rulseh AM. Comparison between postcontrast thin-slice T1-weighted 2D spin echo and 3D T1-weighted SPACE sequences in the detection of brain metastases at 1.5 and 3 T. *Insights Imaging*. (2024) 15:73. 10.1186/s13244-024-01643-6 38483648 PMC10940548

[12] Kwak HS Hwang S Chung GH Song JS Choi EJ. Detection of small brain metastases at 3 T: comparing the diagnostic performances of contrast-enhanced T1-weighted SPACE, MPRAGE, and 2D FLASH imaging. *Clin Imaging*. (2015) 39:571–5. 10.1016/j.clinimag.2015.02.010 25770904

[13] Goncalves Filho ALM Conklin J Longo MGF Cauley SF Polak D Liu W et al. Accelerated post-contrast wave-CAIPI T1 SPACE achieves equivalent diagnostic performance compared with standard T1 SPACE for the detection of brain metastases in clinical 3T MRI. *Front Neurol*. (2020) 11:587327. 10.3389/fneur.2020.587327 33193054 PMC7653188

[14] Gule-Monroe M Chasen N Long JP Kumar VA Shah K Chen M et al. Diagnostic confidence of contrast-enhanced T1-weighted MRI for the detection of brain metastases: 3d FSE versus 3D GRE-based sequences. *AJNR Am J Neuroradiol*. (2024) 46:1231–7. 10.3174/ajnr.A8590 39572196 PMC12152807

[15] Romano A Moltoni G Guarnera A Pasquini L Di Napoli A Napolitano A et al. Single BM versus GBMmultiforme: a VOI-based multiparametric analysis for differential diagnosis. *Radiol Med.* (2022) 127:490–7. 10.1007/s11547-022-01480-x 35316518 PMC9098536

[16] Zhuoni M Xiqi Z. Application and research status of motion sensitization-driven balance technology. *Magn Resonance Imaging.* (2021) 12:121–4. 10.12015/issn.1674-8034.2021.08.029

[17] Jun C Shuhua L Xue Z Chunqing B Mingli H. Application of motion-sensitized driven equilibrium based black blood 3D TSE sequence in the detection of brain metastases. *Magn Reson Imaging*. (2022) 93:145–8. 10.1016/j.mri.2022.08.010 35981693

[18] Chen Zhou ZH Salvador Álvarez E Hilario A Cárdenas Del Carre A Romero Coronado J Lechuga C et al. Improved detection of BM using contrast-enhanced 3D black-blood TSE sequences compared to post-contrast 3D T1 GRE: a comparative study on 1.5-T MRI. *Eur Radiol.* (2025) 35:4267–76. 10.1007/s00330-025-11363-0 39841203

[19] Bae YJ Choi BS Lee KM Yoon YH Sunwoo L Jung C et al. Efficacy of maximum intensity projection of contrast-enhanced 3d turbo-spin echo imaging with improved motion-sensitized driven-equilibrium preparation in the detection of BM. *Korean J Radiol.* (2017) 18:699–709. 10.3348/kjr.2017.18.4.699 28670165 PMC5447646

[20] Lee S Park DW Lee JY Lee YJ Kim T. Improved motion-sensitized driven-equilibrium preparation for 3D turbo spin echo T1 weighted imaging after gadolinium administration for the detection of BM on 3T MRI. *Br J Radiol.* (2016) 89:20150176. 10.1259/bjr.20150176 27187597 PMC5257297

[21] Nagao E Yoshiura T Hiwatashi A Obara M Yamashita K Kamano H et al. 3D turbo spin-echo sequence with motion-sensitized driven-equilibrium preparation for detection of brain metastases on 3T MR imaging. *AJNR Am J Neuroradiol*. (2011) 32:664–70. 10.3174/ajnr.A2343 21292797 PMC7965899

[22] Parillo M Vertulli D Vaccarino F Mallio CA Beomonte Zobel B Quattrocchi CC. The sensitivity of MIPs of 3D contrast-enhanced VIBE T1-weighted imaging for the detection of small brain metastases (≤ 5 mm) on 1.5 tesla MRI. *Neuroradiol J*. (2024) 37:744–50. 10.1177/19714009241260802 38861176 PMC11531042

[23] Sepulveda F Yáñez P Carnevale MD Romero C Castillo M. MIP improves detection of BM. *J Comput Assist Tomogr.* (2016) 40:997–1000. 10.1097/RCT.0000000000000466 27529685

[24] Cheng VWT Soto MS Khrapitchev AA Perez-Balderas F Zakaria R Jenkinson MD et al. VCAM-1-targeted MRI enables detection of brain micrometastases from different primary tumors. *Clin Cancer Res*. (2019) 25:533–43. 10.1158/1078-0432.CCR-18-1889 30389659

[25] Serres S Soto MS Hamilton A McAteer MA Carbonell WS Robson MD et al. Molecular MRI enables early and sensitive detection of BM. *Proc Natl Acad Sci U S A.* (2012) 109:6674–9. 10.1073/pnas.1117412109 22451897 PMC3340084

[26] Cheng VWT de Pennington N Zakaria R Larkin JR Serres S Sarkar M et al. VCAM-1–targeted MRI improves detection of the Tumor-brain interface. *Clin Cancer Res*. (2022) 28:2385–96. 10.1158/1078-0432.CCR-21-4011 35312755 PMC9662863

[27] Nechaeva A Ulitin A Sitovskaya D Yudintceva N Bobkov D Likhomanova R et al. Fluorescence molecular imaging of high-grade gliomas and BM using the RAS70 peptide targeting plasma membrane-bound Hsp70 on tumor cells. *J Neurooncol.* (2025) 176:15. 10.1007/s11060-025-05245-0 41094345 PMC12528191

[28] Tong E McCullagh KL Iv M. Advanced imaging of BM: from augmenting visualization and improving diagnosis to evaluating treatment response. *Front Neurol.* (2020) 11:270. 10.3389/fneur.2020.00270 32351445 PMC7174761

[29] DeBrosse C Nanga RP Bagga P Nath K Haris M Marincola F et al. Lactate chemical exchange saturation transfer (LATEST) imaging in vivo A biomarker for LDH activity. *Sci Rep*. (2016) 6:26794265. 10.1038/srep19517 26794265 PMC4726389

[30] Wang J Weygand J Hwang KP Mohamed AS Ding Y Fuller CD et al. Magnetic resonance imaging of glucose uptake and metabolism in patients with head and neck cancer. *Sci Rep*. (2016) 6:30618. 10.1038/srep30618 27461165 PMC4962090

[31] Herz K Lindig T Deshmane A Schittenhelm J Skardelly M Bender B et al. T1ρ-based dynamic glucose-enhanced (DGEρ) MRI at 3 T: method development and early clinical experience in the human brain. *Magn Reson Med*. (2019) 82:1832–47. 10.1002/mrm.27857 31231853

[32] Wu Y Derks SHAE Wood TC de Blois E van der Veldt AAM Smits M et al. Improved postprocessing of dynamic glucose-enhanced CEST MRI for imaging brain metastases at 3 T. *Eur Radiol Exp*. (2023) 7:78. 10.1186/s41747-023-00390-5 38066225 PMC10709288

[33] Nichelli L Casagranda S Dipasquale O Bensemain M Papageorgakis C Zucchelli M et al. Fluid-suppressed amide proton transfer-weighted imaging outperforms leakage-corrected dynamic susceptibility contrast perfusion in distinguishing progression from radionecrosis in brain metastases. *Cancers*. (2025) 17:1175. 10.3390/cancers17071175 40227736 PMC11987914

[34] Knutsson M Salomonsson T Durmo F Johansson ER Seidemo A Lätt J et al. Differentiation between glioblastoma and solitary brain metastases using perfusion and amide proton transfer weighted MRI. *Front Neurosci*. (2025) 19:1533799. 10.3389/fnins.2025.1533799 39975970 PMC11836003

[35] Kazda T Bulik M Pospisil P Lakomy R Smrcka M Slampa P et al. Advanced MRI increases the diagnostic accuracy of recurrent glioblastoma: single institution thresholds and validation of MR spectroscopy and diffusion weighted MR imaging. *Neuroimage Clin*. (2016) 11:316–21. 10.1016/j.nicl.2016.02.016 27298760 PMC4893011

[36] Sacli-Bilmez B Bas A Erşen Danyeli A Yakicier MC Pamir MN Özduman K et al. Detecting IDH and TERTp mutations in diffuse gliomas using 1H-MRS with attention deep-shallow networks. *Comput Biol Med*. (2025) 186:109736. 10.1016/j.compbiomed.2025.109736 39874812

[37] Nichelli L Cadin C Lazzari P Mathon B Touat M Sanson M et al. Incorporation of Edited MRS into clinical practice may improve care of patients with IDH-mutant glioma. *AJNR Am J Neuroradiol*. (2025) 46:113–20. 10.3174/ajnr.A8413 38997123 PMC11735446

[38] Haddadi Avval A Banerjee S Zielke J Kann BH Mueller S Rauschecker AM. Applications of AI and advanced imaging in pediatric diffuse midline glioma. *Neuro Oncol.* (2025) 27:1419–33. 10.1093/neuonc/noaf058 40037540 PMC12309720

[39] Derks SHAE van der Veldt AAM Smits M. BM: the role of clinical imaging. *Br J Radiol.* (2022) 95:20210944. 10.1259/bjr.20210944 34808072 PMC8822566

[40] She D Yang X Xing Z Cao D. Differentiating hemangioblastomas from brain metastases using diffusion-weighted imaging and dynamic susceptibility contrast-enhanced perfusion-weighted MR imaging. *AJNR Am J Neuroradiol*. (2016) 37:1844–50. 10.3174/ajnr.A4809 27173365 PMC7960479

[41] Zhang L Yao R Gao J Tan D Yang X Wen M et al. An integrated radiomics model incorporating diffusion-weighted imaging and 18F-FDG PET imaging improves the performance of differentiating glioblastoma from solitary BM. *Front Oncol.* (2021) 11:732704. 10.3389/fonc.2021.732704 34527594 PMC8435895

[42] He L Chen M Li H Shi X Qiu Z Xu X. Differentiation between high-grade gliomas and solitary BM based on multidiffusion MRI model quantitative analysis. *Front Oncol.* (2024) 14:1401748. 10.3389/fonc.2024.1401748 39469636 PMC11513521

[43] Pope WB. Brain metastases: neuroimaging. *Handb Clin Neurol*. (2018) 149:89–112. 10.1016/B978-0-12-811161-1.00007-4 29307364 PMC6118134

[44] Breckwoldt M Bendszus M. Zerebrale MR-Bildgebung beim malignen Melanom [Cerebral MR imaging of malignant melanoma]. *Radiologe.* (2015) 55:113–9. 10.1007/s00117-014-2761-0 25589420

[45] Sun L Chen J Liu X Zhu H Dong H Tao G et al. Characterization of brain metastases from lung cancer using percentagewise quantification of intratumoral susceptibility signals. *Quant Imaging Med Surg*. (2025) 15:5373–83. 10.21037/qims-24-1505 40606379 PMC12209704

[46] Su Y Wang J Guo J Liu X Yang X Cheng R et al. Bi-exponential diffusion-weighted imaging for differentiating high-grade gliomas from solitary BM: a VOI-based histogram analysis. *Sci Rep.* (2024) 14:31909. 10.1038/s41598-024-83452-x 39738411 PMC11685987

[47] Mehrabian H Detsky J Soliman H Sahgal A Stanisz GJ. Advanced magnetic resonance imaging techniques in management of brain metastases. *Front Oncol*. (2019) 9:440. 10.3389/fonc.2019.00440 31214496 PMC6558019

[48] Park SY Mosci C Kumar M Wardak M Koglin N Bullich S et al. Initial evaluation of (4S)-4-(3-[18F]fluoropropyl)-L-glutamate (FSPG) PET/CT imaging in patients with head and neck cancer, colorectal cancer, or non-Hodgkin lymphoma. *EJNMMI Res*. (2020) 10:100. 10.1186/s13550-020-00678-2 32857284 PMC7455665

[49] Stopa BM Juhász C Mittal S. Comparison of amino acid PET to advanced and emerging MRI techniques for neurooncology imaging: a systematic review of the recent studies. *Mol Imaging.* (2021) 2021:8874078. 10.1155/2021/8874078 34194287 PMC8205602

[50] Pruis IJ van Dongen GAMS Veldhuijzen van Zanten SEM. The added value of diagnostic and theranostic PET imaging for the treatment of CNS tumors. *Int J Mol Sci*. (2020) 21:1029. 10.3390/ijms21031029 32033160 PMC7037158

[51] Cheng X Hou G Zheng R Zhang J Wang X. Detection of small brain metastases by 18 F-Thretide PET/CT in clear cell renal cell carcinoma. *Clin Nucl Med*. (2025) 50:183–5. 10.1097/RLU.0000000000005523 39480242

[52] Galldiks N Kocher M Ceccon G Werner JM Brunn A Deckert M et al. Imaging challenges of immunotherapy and targeted therapy in patients with brain metastases: response, progression, and pseudoprogression. *Neuro Oncol*. (2020) 22:17–30. 10.1093/neuonc/noz147 31437274 PMC6954406

[53] Rosen J Werner JM Ceccon GS Rosen EK Wollring MM Stetter I et al. MRI and 18F-FET PET for multimodal treatment monitoring in patients with brain metastases: a cost-effectiveness analysis. *J Nucl Med*. (2024) 65:838–44. 10.2967/jnumed.123.266687 38664020

[54] Mahmoud O Püllen L Umutlu L Szarvas T Fendler WP Ting S et al. Multitracer comparison of gold standard PSMA-PET/CT with 68Ga-FAPI and 18F-FDG in high-risk prostate cancer: a proof-of-concept study. *Eur J Nucl Med Mol Imaging*. (2025) 52:4860–9. 10.1007/s00259-025-07352-6 40423777

[55] Lim W Acker G Hardt J Kufeld M Kluge A Brenner W et al. Dynamic 18F-FET PET/CT to differentiate recurrent primary brain tumor and BM from radiation necrosis after single-session robotic radiosurgery. *Cancer Treat Res Commun.* (2022) 32:100583. 10.1016/j.ctarc.2022.100583 35688103

[56] Aggarwal A Aggarwal AK Prakash S Vile DJ Aggarwal A. Narrow interval dual phase 18F-FDG PET/CT: A practical approach for distinguishing tumor recurrence from radiation necrosis in BM. *Medicine.* (2024) 103:e37789. 10.1097/MD.0000000000037789 38701250 PMC11062716

[57] Rehm J Winzer R Notni J Hempel S Distler M Folprecht G et al. Concomitant metastatic head-and-neck cancer and pancreatic cancer assessed by αvβ6-integrin PET/CT using 68Ga-Trivehexin: incidental detection of a brain metastasis. *Eur J Nucl Med Mol Imaging*. (2024) 51:3469–71. 10.1007/s00259-024-06750-6 38771514 PMC11368998

[58] Wang P Li T Zhuang H Li F Jing H. 18 F-MFBG PET/CT and MRI in identifying BM in a posttreatment neuroblastoma patient. *Clin Nucl Med.* (2024) 49:600–3. 10.1097/RLU.0000000000005224 38584349

[59] Poterszman N Somme L Bund C Hutt E Somme FA. Hangover under 177 Lu-PSMA-617 therapy : a red flag for brain 68 Ga-PSMA-11 PET/MRI? *Clin Nucl Med.* (2024) 49:582–3. 10.1097/RLU.0000000000005126 38389216

[60] Tutic-Sorrentino L Cazzaniga S Feldmeyer L Benzaquen M. Positron emission tomography-CT vs. brain MRI for the detection of cerebral metastases of melanoma: a 5-year retrospective study. *Clin Exp Dermatol.* (2024) 49:1179–85. 10.1093/ced/llae129 38624009

[61] Zhang B Zhu J Xu R Zou L Lian Y Xie X et al. A combined model integrating radiomics and deep learning based on multiparametric MRI for classification of BM. *Acta Radiol.* (2025) 66:24–34. 10.1177/02841851241292528 39552295

[62] van de Weijer T Broen MPG Moonen RPM Hoeben A Anten M Hovinga K et al. The Use of 18F-FET-PET-MRI in neuro-oncology: the best of both worlds-a narrative review. *Diagnostics*. (2022) 12:1202. 10.3390/diagnostics12051202 35626357 PMC9140561

[63] Øen SK Johannessen K Pedersen LK Berntsen EM Totland JA Johansen H et al. Diagnostic Value of 18 F-FACBC PET/MRI in BM. *Clin Nucl Med.* (2022) 47:1030–9. 10.1097/RLU.0000000000004435 36241129 PMC9653108

[64] Shin I Kim H Ahn SS Sohn B Bae S Park JE et al. Development and validation of a deep learning-based model to distinguish glioblastoma from solitary brain metastasis using conventional MR images. *AJNR Am J Neuroradiol*. (2021) 42:838–44. 10.3174/ajnr.A7003 33737268 PMC8115383

[65] Cui L Yu L Shao S Zuo L Hou H Liu J et al. Improving differentiation of hemorrhagic brain metastases from non-neoplastic hematomas using radiomics and clinical feature fusion. *Neuroradiology*. (2025) 67:1455–68. 10.1007/s00234-025-03590-5 40131431

[66] Xia X Tan Q Du W Gou Q. Radiomics-based machine learning for differentiating lung squamous cell carcinoma and adenocarcinoma using T1-enhanced MRI of BM. *Front Oncol.* (2025) 15:1599853. 10.3389/fonc.2025.1599853 40772029 PMC12325419

[67] Linyan X Jie C Kexuan Z Houquan C Chaoyi Q Xiaosong Y et al. A multimodal fusion network based on variational autoencoder for distinguishing SCLC brain metastases from NSCLC brain metastases. *Med Phys*. (2025) 52:e17816. 10.1002/mp.17816 40318172

[68] Meißner AK Gutsche R Galldiks N Kocher M Jünger ST Eich ML et al. Radiomics for the noninvasive prediction of the BRAF mutation status in patients with melanoma BM. *Neuro Oncol.* (2022) 24:1331–40. 10.1093/neuonc/noab294 34935978 PMC9340614

[69] Deng F Xiao G Tanzhu G Chu X Ning J Lu R et al. Predicting survival rates in BM patients from NSCLC using radiomic signatures associated with tumor immune heterogeneity. *Adv Sci.* (2025) 12:e2412590. 10.1002/advs.202412590 39840456 PMC11904944

[70] Premachandran S Shreshtha I Venkatakrishnan K Das S Tan B. Detection of brain metastases from blood using Brain nanoMET sensor: extracellular vesicles as a dynamic marker for metastatic brain tumors. *Biosens Bioelectron*. (2025) 269:116968. 10.1016/j.bios.2024.116968 39586755

[71] Zuccato JA Patil V Mansouri S Voisin M Chakravarthy A Shen SY et al. Cerebrospinal fluid methylome-based liquid biopsies for accurate malignant brain neoplasm classification. *Neuro Oncol*. (2023) 25:1452–60. 10.1093/neuonc/noac264 36455236 PMC10398815

[72] Straub S Schneider TM Emmerich J Freitag MT Ziener CH Schlemmer HP et al. Suitable reference tissues for quantitative susceptibility mapping of the brain. *Magn Reson Med.* (2017) 78:204–14. 10.1002/mrm.26369 27529579

[73] Doniza L Lee M Blumenfeld-Katzir T Artzi M Ben-Bashat D Aizenstein O et al. Noise propagation and MP-PCA image denoising for high-resolution quantitative $R_2^∧^{\rm{*}}$, $T_2^∧^{\rm{*}}$, and magnetic susceptibility mapping (QSM). *IEEE Trans Biomed Eng*. (2025) 72:3277–87. 10.1109/TBME.2025.3566561 40315096

